# Supplementary material for: Risk factors for sacrococcygeal pilonidal sinus: a systematic review and meta-analysis supplemented by genetic causal assessment
Source: Front Surg. 2026 Jan 7;12:1718589. doi: 10.3389/fsurg.2025.1718589 (PMC12819706; doi:10.3389/fsurg.2025.1718589)
Supplement: Supplementary file 2 [file Datasheet2.zip › Supplementary Data 2/MR_pipeline_p5e-8/forest_plots/ukb-saige-272.1.finngen_R12_L12_PILONIDALCYST.pdf]

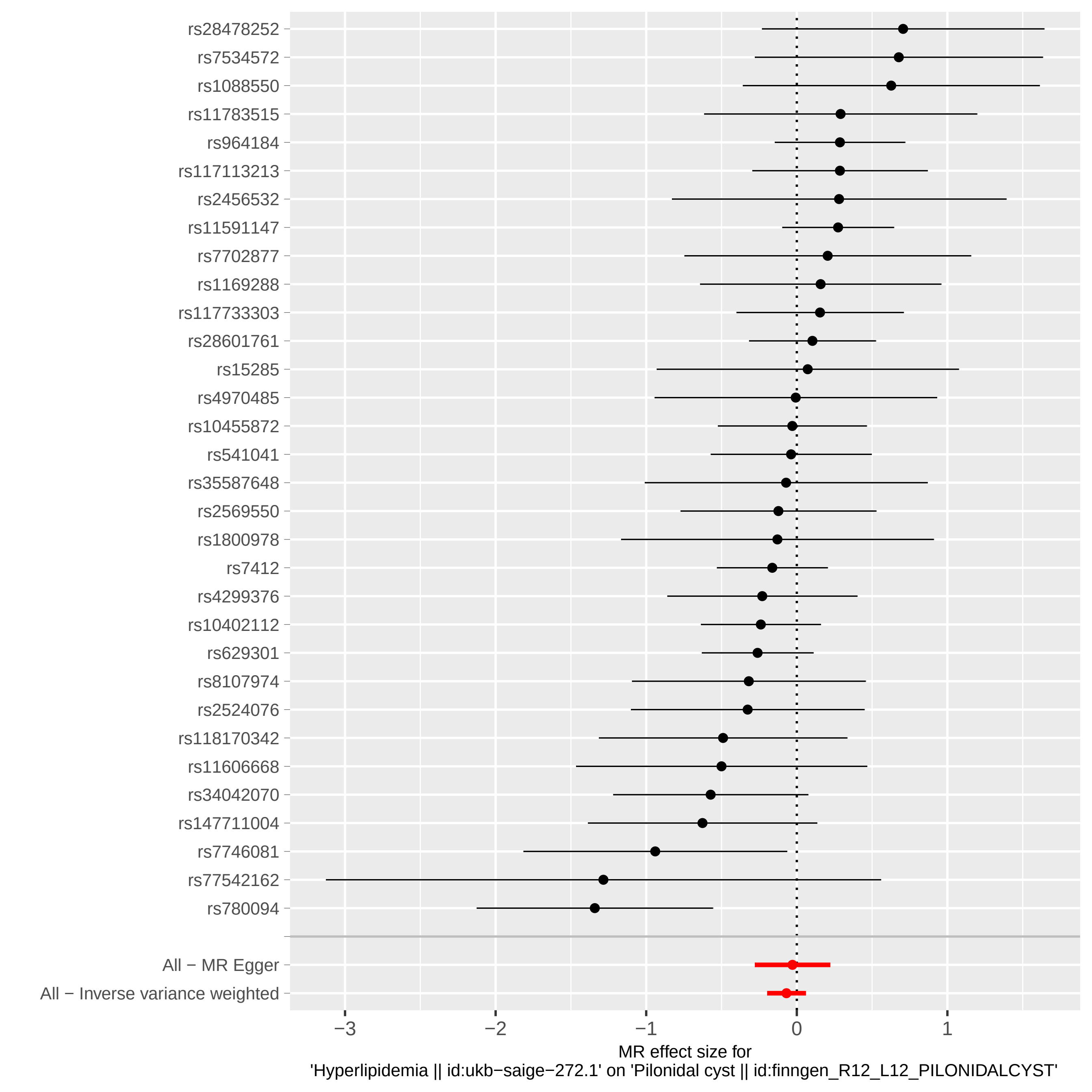

rs28478252
rs7534572
rs1088550
rs11783515
rs964184
rs117113213
rs2456532
rs11591147
rs7702877
rs1169288
rs117733303
rs28601761
rs15285
rs4970485
rs10455872
rs541041
rs35587648
rs2569550
rs1800978
rs7412
rs4299376
rs10402112
rs629301
rs8107974
rs2524076
rs118170342
rs11606668
rs34042070
rs147711004
rs7746081
rs77542162
rs780094
All − MR Egger
All − Inverse variance weighted
−3
−2
−1
0
1
MR effect size for
'Hyperlipidemia || id:ukb−saige−272.1' on 'Pilonidal cyst || id:finngen_R12_L12_PILONIDALCYST'
